# The Role of Physical Activity in the Relationship between Satisfaction with Life and Health-Related Quality of Life in School-Age Adolescents

**DOI:** 10.3390/bs11090121

**Published:** 2021-09-03

**Authors:** Santos Villafaina, Miguel Ángel Tapia-Serrano, Mikel Vaquero-Solís, Juan Luis León-Llamas, Pedro Antonio Sánchez-Miguel

**Affiliations:** 1 Physical Activity and Quality of Life Research Group (AFYCAV), Faculty of Sport Sciences, University of Extremadura, Avd. de la Universidad S/N, 10003 Cáceres, Spain; svillafaina@unex.es (S.V.); leonllamas@unex.es (J.L.L.-L.); 2 Department of Didactics of Music, Plastic and Body Expression, Teacher Training College, University of Extremadura, Avd. de la Universidad S/N, 10003 Cáceres, Spain; mivaquero89@gmail.com (M.V.-S.); pesanchezm@unex.es (P.A.S.-M.)

**Keywords:** health-related quality of life, exercise, adolescence

## Abstract

(1) Background: Adolescence is a critical stage in the development of healthy habits. In this regard, physical activity has emerged as a useful tool to improve satisfaction with life and health-related quality of life in adolescents. Therefore, the aim of the present study was to examine the mediating role of physical activity in the relationship between satisfaction with life and health-related quality of life in adolescent boys and girls. Also, we aimed to investigate the differences between sexes in the HRQoL, physical activity level, and satisfaction with life. (2) Methods: A total of 297 adolescents, ranging in age from 11 to 12 years (11.46 ± 1.63), participated in this cross-sectional study. The Satisfaction with life scale, Physical activity Questionnaire for Adolescents and the KIDSCREEN-10 questionnaires were employed. (3) Results: The estimated indirect effect showed that physical activity level was a mediator of the positive effect of satisfaction with life on health-related quality of life (β = 0.105, 95% CI = 0.031; 0.202). However, the index of moderated mediation showed that sex is not a significant moderator of the mediating role of physical activity in the relationship between satisfaction with life and HRQoL (β = −0.033, 95% CI = −0.023, 0.136). Furthermore, significant differences in satisfaction with life were found, with girls manifesting lower values (*p*-value = 0.026). (4) Conclusion: This study shows the importance of physical activity during adolescence and the association of this behavior with the health-related quality of life of adolescents.

## 1. Introduction

Adulthood health-related behaviors are predicted by those which adolescence can develop. Thus, adolescence is considered as a critical stage in the development of healthy habits [1,2]. However, a decrease in the physical activity levels and an increase in sedentary activities can be observed during adolescence [3]. This is relevant since these unhealthy habits can lead to an increase in being overweight and obesity, as well as that those adolescents with healthy lifestyles reported higher health related quality of life (HRQoL) [4] (a multi-dimensional concept that includes domains related to physical, mental, emotional, and social functioning). Furthermore, adolescence plays a relevant role in satisfaction with life [5] and mental well-being [6]. Nevertheless, differences can be observed among sex. In this regard, girls tend to be less active [7,8] than boys, as well as to report lower levels of satisfaction with life [9].

The COVID-19 pandemic has caused children and adolescents to face massive changes in their daily lives such as school closures, confinement, or social distancing, which can burden them substantially [10,11]. This has increased the problem of sedentary lifestyles in children and adolescents, with 81% of students physically inactive before the pandemic [12], reducing active leisure activities while increasing screen recreational time [13,14]. This has burdened children and adolescent’s health substantially [10,11], reducing their HRQoL [15] and satisfaction with life [16].

### 1.1. Physical Activity and Health-Related Quality of Life

Physical activity has emerged as a useful tool to maintain a healthy lifestyle [17]. In this regard, several studies have pointed out the relationship between physical activity and HRQoL [18,19,20,21,22]. Thus, more than sixteen articles in the last year and a half have explored this relationship. In this regard, a previous study showed that physical activity levels can predict HRQoL in children as well as that physical activity is the main behavior in the prediction of the quality of life for a population of school adolescents [20]. Furthermore, improvements in cardiorespiratory fitness may be useful to improve HRQoL [18,19]. Strengthening the relationship between level of physical activity and HRQoL, we can find different articles exploring sedentary behaviors and their relation to HRQoL [21]. In this regard, previous studies showed that adolescents who had longer exposure and lower physical activity levels reported lower HRQoL [22].

Moreover, previous studies have been focused on the impact of physical activity volume and intensity on the improvements in HRQoL. Regarding physical activity volume, previous studies found that the higher the physical activity level, the higher the HRQoL [23]. Regarding physical activity intensity and improvement in HRQoL, a previous study showed that children who spent more time practicing moderate PA were associated with higher HRQoL scores [24]. In the same vein, a study that explore the commitment of physical activity recommendations found that meeting combinations of screen time with moderate-to-vigorous physical activity were related to higher HRQoL [25]. However, a previous study found that light intensity physical activity was associated with higher HRQoL in females whereas, in males, moderate and vigorous PA levels were associated with higher HRQoL values [26].

Due to the relationship between physical activity and HRQoL in children and adolescents, a previous study has been focused on the influence of school-hours physical activity and quality of life [27]. This study found a positive association between children’s school-hours physical activity and HRQoL [27]. This result reinforces the usefulness of school-based physical activity programs to improve HRQoL.

### 1.2. Physical Activity and Satisfaction with Life

Although multiple studies have shown a direct relationship between physical activity level and satisfaction with life [5,28,29], the relationship between physical activity and satisfaction with life has not been so widely studied.

Higher levels of screen time and lower levels of physical activity were associated with lower satisfaction with life [30]. Nevertheless, increasing physical activity is more crucial than emphasizing reducing screen time in improving the satisfaction with life of children and adolescents [31]. Furthermore, Chmelík, et al. [32] showed that adolescents with higher satisfaction with life had higher physical activity levels as well as that those who spent more time engaged in physical activity showed higher satisfaction with life [5].

Regarding physical activity intensity, three studies [32,33] showed that the strongest associations between satisfaction with life and physical activity were found in those adolescents who participated in vigorous physical activity. However, as previously found for the HRQoL [26], boys and girls differently responded to physical activity. In this regard, for boys, vigorous physical activity can be regarded as the predictor of better satisfaction with life, while, for girls, moderate-to-vigorous physical activity might be considered as a risk factor for lower life satisfaction [34].

Due to the significant association between physical activity enrolment and satisfaction with life, a previous study has encouraged public investments to promote adherence to physical activity in adolescents [35].

### 1.3. Satisfaction with Life and Health-Related Quality of Life

Satisfaction with life and HRQoL are considered to be related to achieving a healthy lifestyle [36]. Thus, the relationship between satisfaction with life and HRQoL has been shown in previous studies [37,38]. Satisfaction with life implies acceptance of one’s life circumstances in relation to the subjective judgment of their life situation in relation to one’s own expectations [36]. Thus, satisfaction with life is considered by previous studies as a subjective and cognitive assessment of one’s quality of life [36,39]. Nevertheless, HRQol would indicate a general and constant state of well-being [40], which covers broad domains including psychological, spiritual, physical, economical, and social well-being [41]. Regarding the physical domain of the HRQoL, a previous study found that, together with the psychological domain, they were significant predictors of satisfaction with life [42]. Furthermore, a previous study found a mediating and predictive role of subjective happiness in HRQoL [43].

A previous study listed the factors affecting individuals’ life satisfaction in the mental, physical, and social spheres: feeling well physically, taking pleasure in life, consistency at the matter of reaching goals, social relationships, finding life meaningful, positive individual identity, and economical security [44]. In this regard, physical activity can help to improve most of these factors. Furthermore, since HRQoL is a multi-dimensional concept that includes physical, mental, and social domains, improvements in satisfaction with life would result in changes in the HRQoL. Thus, physical activity might have a mediating role in the relationship between satisfaction with life and HRQoL.

### 1.4. Objectives and Novelty of the Study

Taking into account the previous analyzed studies, there are several studies which investigate the relationship between physical activity level and HRQoL, and satisfaction with life. However, there is a lack of studies which investigate the mediating role of physical activity in the relationship between satisfaction with life and HRQoL in adolescent boys and girls. Therefore, the present study aimed: (1) to evaluate the mediating role of physical activity between satisfaction with life and HRQoL; (2) to investigate the moderating role of sex on the mediating role of physical activity in the relationship between satisfaction with life and HRQoL; (3) to investigate the differences between sex in the HRQoL, physical activity level, and satisfaction with life.

This study, to the best of our knowledge, would be the first which analyzed the mediating role of physical activity in the relationship between satisfaction with life and HRQoL in adolescents. Furthermore, it would be also the first one which analyzed this relationship while taking into account the adolescents’ sex.

## 2. Materials and Methods

### 2.1. Participants

Sample size calculation estimated a minimum of 278 participants to reach a moderate effect size. Finally, a total of 297 adolescents, ranging in age from 11 to 12 years (11.46 ± 1.63), participated in this cross-sectional study. The PASS-21 software (NCSS, Kaysville, UT, USA) estimated that a sample size of 297 achieves 94% power to detect a mediation effect, taking into account the product of the regression coefficients and the standard deviation of the mediator, and the independent and dependent variables.

A researcher contacted 10 educational centres of Extremadura (Spain). The educational centres were asked to disseminate the information to the teachers of the centre. Nevertheless, due to COVID-19 restrictions, six educational schools (primary or high schools) decided to participate. Another researcher administered and explained the questionnaires in the classrooms. All queries were solved by the researcher or the teacher, who was also present at the classroom.

The study was conducted in accordance with the Declaration of Helsinki, and the protocol was approved by the Bioethics and Biosafety Committee of the University of Extremadura (89/2016). Parents or legal guardians signed written informed consent prior to participation in the study.

### 2.2. Measures

*Sociodemographic characteristics.* Participants reported age, sex, and socioeconomic status (SES). Participant’s SES were obtained using a modified version of the Family Affluence Scale-II (FAS II; [45]). Participants were asked if they have a room for themselves, if they have internet access at home, the number of personal computers at home, and the number of cars.

*Satisfaction with life.* Satisfaction with life was measured using the satisfaction with life scale (SWLS) [46]. This study employed the Spanish version of the SWLS [47]. The internal consistency of this scale is 0.84 according to Cronbach’s Alpha. This scale has 5 items, with a 5-point Likert scale, ranging from 1 (totally disagree) to 5 (totally agree). The summary of the items is used to calculate the total SWLS score.

*Physical activity.* Physical activity was assessed with the Physical Activity Questionnaire for Adolescents (PAQ-A) [48], using the Spanish version [49]. Internal consistency of the Spanish version showed a Cronbach’s Alpha = 0.65. This questionnaire evaluated the self-reported physical activity level. This is a self-administered, 7-day recall instrument, with 9 items scored on a 5-point scale. To calculate a physical activity score, the frequency of participation in a list of activities such as physical activity in Physical Education lessons, during the school break, at lunchtime, right after school, and evenings, as well as during the last weekend were asked. The mean of item 1 to 8 is used to calculate the final PAQ-A activity summary score.

*Health-related Quality of life.* HRQoL was measured with the Spanish version of the KIDSCREEN-10 questionnaire [50]. Internal consistency of KIDSCREEN-10 total score showed a Cronbach’s Alpha = 0.82. This scale comprises a total of 10 items in a 5-point Likert scale ranging from 1 (never) to 5 (always). Responses were coded so that higher values indicated higher HRQoL. Responses were summed and Rasch person parameters were assigned to each possible sum score. The person parameters were transformed into values with a mean of 50 and a standard deviation (SD) of approximately 10 [51].

### 2.3. Statistical Analysis

The SPSS statistical package (version 22.0; SPSS, Inc., Chicago, IL, USA) was used to conduct the analyses. Categorical variables are presented using frequencies and percentages, and continuous variables are presented using means and standard deviations. We applied covariance analysis adjusted by sex. Taking into account the results of Kolgomorov–Smirnov and Shapiro–Wilk tests, tests for independent samples or Mann–Whitney U were conducted to investigate differences between sex in HRQoL, satisfaction with life, and physical activity level. Effect sizes, Cohens’D or r, were calculated if p-value was obtained from a parametric or a non-parametric test respectively.

In order to test the possible mediating role of physical activity between satisfaction with life and HRQoL, a simple mediation analysis was designed using the “model 4” of the PROCESS macro for SPSS provided by Hayes [52]. In this model, satisfaction with life will be the independent variable (X variable), physical activity will be the mediating variable (M variable), and health-related quality of life will be the dependent variable (Y variable). We also included age as a covariate variable due to the possible impact of age on health-related quality of life [53]. This analysis would allow us to estimate the indirect effect of physical activity level as a mediator between satisfaction with life and HRQoL.

Furthermore, a moderated-mediation analysis was conducted using the “model 58” of the PROCESS macro for SPSS [52] to test the moderating impact of sex on satisfaction with life and physical activity relationship, and in the relationship between physical activity level and HRQoL. For this analysis, satisfaction with life was entered into the model as the independent variable (X variable), the physical activity level was entered into the model as the mediator (M variable), sex (dummy-coded; 0 = girls, 1 = boys) was included as the moderator (W variable), and HRQoL was specified as the outcome variable (Y variable). Age was also included as a covariate variable due to the possible impact of age on health-related quality of life [53]

The PROCESS macro for SPSS (IBM, Chicago, IL, USA) [52] was used to perform the analyses. The mediation hypotheses were tested using the bias-corrected bootstrap method with 5000 samples to calculate confidence intervals (95%). Significance was considered when the confidence interval did not cross zero.

## 3. Results

Table 1 shows the descriptive characteristics of participants. They had a mean age of 11.46 (1.63) years old. Most of them had a room for themselves (83.2%) as well as internet access at home 97.9%. Adolescents had a mean HRQoL score of 48.08 (0.62), a physical activity level of 2.79 (0.62), and a satisfaction with life of 19.30 (3.98).

**Figure 1:**
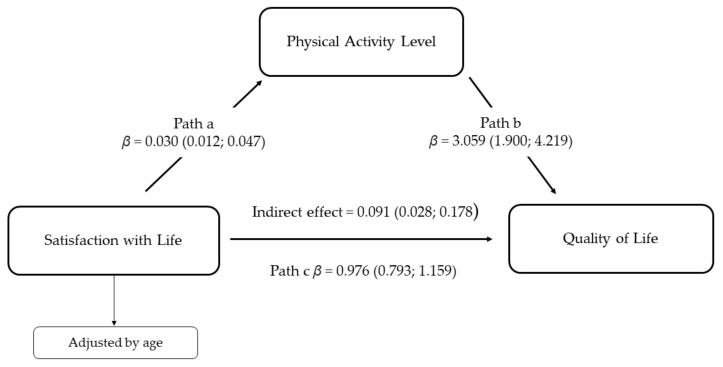
Simple mediation analysis of the effect of satisfaction with life on HRQoL mediated by physical activity level. This model was adjusted by age and the number of bootstrap samples was 5000. Path a = association between satisfaction with life and physical activity level; Path b = association between physical activity level and HRQoL; path c = direct effect of the satisfaction with life and HRQoL.

Figure 1 shows the mediation model adjusted by age. Path a showed a significant direct association between satisfaction with life and physical activity level (β = 0.030, 95% CI = 0.012; 0.047). Furthermore, age (the covariate included in the model) showed a significant influence on physical activity (β = −0.063, 95% CI = −0.106; −0.019). Path b showed a significant direct association between physical activity level and HRQoL in adolescents (β = 3.059, 95% CI = 1.900; 4.219). Moreover, age also showed a significant influence on HRQoL (β = −0.674, 95% CI = −1.119; −0.230). The direct effect, path c, showed a significant association between satisfaction with life and HRQoL (β = 0.976, 95% CI = 0.7930; 1.159). Furthermore, the estimated indirect effect showed that physical activity level is a mediator of the positive effect of satisfaction with life on HRQoL (β = 0.091, 95% CI = 0.028; 0.178).

**Figure 2:**
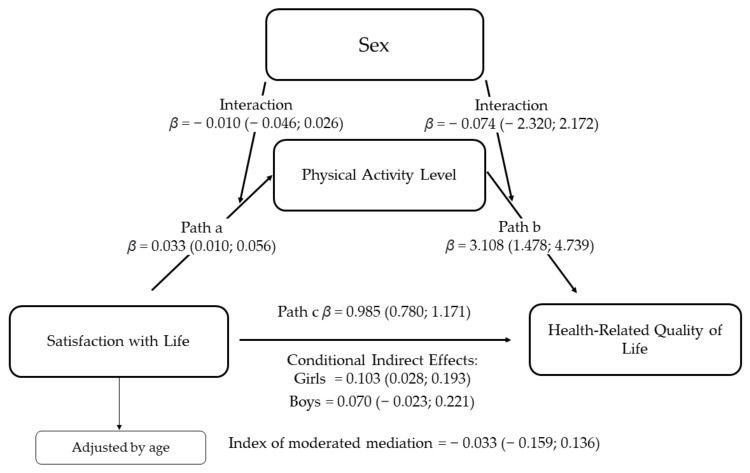
Moderated-mediation analysis of the relation between satisfaction with life and HRQoL mediated by physical activity level and moderated by sex. This model was adjusted by age and the number of bootstrap samples was 5000. Path a = association between satisfaction with life and physical activity level; Path b = association between physical activity level and HRQoL; Path c = direct effect of the satisfaction with life and HRQoL.

Figure 2 show the moderated-mediated analysis. Sex did not moderate the interaction between satisfaction with life and physical activity level (β = −0.010, 95% CI = −0.046; 0.026) nor the relationship between physical activity and HRQoL (β = 0.074, 95% CI = −2.320; 2.172). The covariate, age, also showed significant in physical activity (β = −0.063, 95% CI = −0.106; −0.019) and HRQoL (β = −0.680, 95% CI = −1.127; −0.234). The conditional indirect effect between satisfaction with life and HRQoL through physical activity was significant for adolescent girls (β = 0.103, 95% CI = 0.028; 0.193), whereas it did not reach the significance level for adolescents’ boys (β = 0.070, 95% CI = −0.023; 0.221). However, the confidence interval for the index of moderated mediation did not show a significant effect of sex as a moderator (β = −0.033, 95% CI = −0.023, 0.136). Thus, it cannot be said with 95% confidence that the indirect effect depends on adolescents’ sex.

Table 2 shows the differences between girls and boys in the HRQoL, satisfaction with life, and physical activity level. Boys showed higher levels of satisfaction with life compared to girls (*p* < 0.05). However, no significant differences were found for HRQoL and activity levels (*p* > 0.05) between girls and boys.

## 4. Discussion

This study aimed to investigate the mediating role of physical activity level on the relationship between satisfaction with life and HRQoL in adolescents. Furthermore, this study also investigated sex as a moderator of the mediating role of physical activity in the relationship between satisfaction with life and HRQoL. Also, this study aimed to investigate if there are differences between girls and boys in the HRQoL, satisfaction with life, and physical activity level. The main finding of the present study was that physical activity has a mediating role in the relationship between satisfaction with life and HRQoL. However, sex is not a significant moderator of the mediating role of physical activity in the relationship between satisfaction with life and HRQoL. Moreover, this study also confirms that adolescent girls have less satisfaction with life than adolescent boys.

The first objective of the study was to investigate the mediating role of physical activity in the relationship between satisfaction with life and HRQoL. Our results showed a significant mediating role of physical activity in the relation between satisfaction with life and HRQoL. Moreover, results showed a direct relationship between physical activity and HRQoL. This relationship has been found in previous studies [54], where a higher level of physical activity was associated with an increased HRQoL [4]. Furthermore, a previous study reported a dose–response association between moderate to vigorous physical activity and HRQoL, where an increase in physical activity was associated with additional HRQoL benefits [55]. This finding is supported by previous studies [4,56,57]. Also, this association has been corroborated by longitudinal and interventional studies in adolescents [57,58]. This showed the importance of physical activity on the maintenance and improvements of HRQoL. Interestingly, a previous study showed that body mass index has a relevant role in this association. Nevertheless, another study found that, irrespectively of their body mass index, less physically active children had lower HRQoL [59]. This could indicate that active children and adolescents, just by practicing physical activity, report a better HRQoL. Taking into account that both HRQoL questionnaires, such as KIDSCREEN-10, ask about physical, social, and mental dimensions and the effect of physical activity on these dimensions [60,61], it is completely justified that this is significant relationship. This is of special importance nowadays due to the negative impact of the COVID-19 pandemic on the HRQoL of children and adolescents. Thus, physical activity interventions are encouraged to ameliorate the negative impact of the pandemic on the HRQoL of adolescents.

The second objective of this study was to explore the role of sex as a moderator. Results of the moderated-mediation analysis showed that age is not a moderator. However, the conditional indirect effect between satisfaction with life and HRQoL through physical activity was significant for adolescent girls, whereas it did not reach the significance level for adolescent boys. This result would be supported by a previous study which indicated that a higher level of physical activity longitudinally predicted a greater life satisfaction in adolescent girls after the one-year follow-up, but not in boys [62]. This is relevant since a previous study showed that girls tend to be less active than boys, as well as to report a lower level of satisfaction with life [9]. Regarding physical inactivity in adolescent girls, a previous study hypothesized that gender stereotypes could cause rejection of some physical activities since they are perceived as masculine [63]. Therefore, future interventions should be focused on this aspect, promoting physical activity for girls due to the impact on satisfaction with life in adolescent girls.

In our study, a significant direct effect of satisfaction with life on HRQoL has been found in adolescents, independently of their sex. Moreover, we found that satisfaction with life had a positive effect on HRQoL. However, a previous study indicated that satisfaction with life and HRQoL are similar interdependent concepts, arguing that satisfaction with life is a global assessment of HRQoL [64]. Furthermore, our study also showed a positive relationship between physical activity level and satisfaction with life. This is in line with previous studies where this relationship was also observed [65,66,67].

The third objective of this study was to investigate the differences among sexes in the HRQoL, satisfaction with life, and physical activity level. In this regard, adolescence is considered as a critical stage in the development of healthy habits with critical changes in their lifestyles [1,2]. In this regard, differences between girls and boys were found in satisfaction with life, with girls reporting lower levels. This is in line with previous studies where this difference was observed [9,68]. Studies also reported that males have higher levels of HRQoL [53] and physical activity [8,69]. Although the significance level in this study was not reached, values were also higher in boys than in girls.

This study has some limitations that should be acknowledged. First, the sample was recruited from a region of Spain, and therefore results cannot be extrapolated to another sociodemographic context. Furthermore, future research should include further samples in order to analyse the mediating role of physical activity in different subgroups of adolescents.

## 5. Conclusions

Physical activity has a significant mediating role on the relationship between satisfaction with life and HRQoL. According to the moderated-mediation analysis, sex did not show a significant effect as a moderator in the mediating role of physical activity in the relationship between satisfaction with life and HRQoL. However, the conditional indirect effect between satisfaction with life and HRQoL through physical activity was significant for adolescent girls, whereas it did not reach the significance level for adolescent boys. Furthermore, adolescent girls showed a lower significant level of satisfaction with life than adolescent boys. Therefore, future studies should be specifically focused on promoting physical activity interventions for girls in order to increase their satisfaction with life.

## Figures and Tables

**Table 1:** Descriptive characteristics of participants.

Participants	Mean (SD) or Number (%)	Girls	Boys
Number of participants	297	152	145
Age (age)	11.46 (1.63)	11.57 (1.70)	11.32 (1.54)
Sex [Male (Female)]	145 (152)	-	-
Modified version of the Family Affluence Scale-II			
Room for him/herself	247 (83.2%)	132 (86.8%)	115 (79.3%)
Internet access at home (yes)	291 (97.9%)	148 (97.4%)	143 (98.6%)
Number of personal computers at home			
None	12 (4%)	3 (2%)	9 (6.2%)
One	87 (29.3%)	46 (30.3%)	41 (28.3%)
Two or three	161 (54.3%)	83 (54.5%)	78 (53.8%)
More than three	37 (12.4%)	20 (13.2%)	17 (11.7%)
Number of cars			
None	1 (0.3%)	1 (0.7%)	0 (0%)
One	58 (19.5%)	31 (20.4%)	27 (18.6%)
Two or three	230 (77.5%)	116 (76.3%)	114 (78.6%)
More than three	8 (2.7%)	4 (2.7%)	4 (2.8)
HRQoL score ^a^	48.08 (0.62)	47.62 (7.84)	48.88 (7.66)
Physical activity level ^b^	2.79 (0.62)	2.75 (0.62)	2.85 (0.62)
Sport and activity list	1.64 (0.45)	1.62 (0.45)	1.66 (0.45)
Physical activity during clasess	4.02 (0.92)	4.03 (0.88)	4.03 (0.96)
At lunch	1.70 (1.13)	1.66 (1.12)	1.77 (1.15)
After school	3.06 (1.26)	3.07 (1.24)	3.08 (1.29)
Evenings	3.13 (1.17)	3.12 (1.08)	3.16 (1.25)
Weekends	3.11 (1.10)	3.05 (1.11)	3.19 (1.09)
Statements	2.65 (1.15)	2.49 (1.14)	2.82 (1.14)
Weekly activity	3.06 (0.74)	3.02 (0.69)	3.13 (0.78)
Satisfaction with life ^c^	19.30 (3.98)	18.73 (4.33)	19.95 (3.42)
In most ways my life is close to my ideal	3.36 (1.10)	3.29 (1.10)	3.42 (1.11)
The conditions of my life are excellent	3.98 (1.07)	3.78 (1.11)	4.21 (0.97)
I am satisfied with my life	4.27 (1.06)	4.08 (1.19)	4.51 (0.80)
So far I have gotten the important things I want in life	3.97 (0.99)	3.90 (1.08)	4.06 (0.88)
If I could live my life over, I would change almost nothing	3.71 (1.37)	3.68 (1.38)	3.75 (1.35)

Responses were summed and Rasch person parameters were assigned to each possible sum score. The person parameters were transformed into values with a mean of 50 and a standard deviation (SD) of approximately 10. The mean of item 1 to 8 is used to calculate the final PAQ-A activity summary score. The summary of the items is used to calculate the total SWLS score. Path a = association between satisfaction with life and physical activity level; Path b = association be-tween physical activity level and HRQoL; Path c = direct effect of the satisfaction with life and HRQoL.

**Table 2:** Differences between girls and boys in HRQoL, satisfaction with life, and physical activity level.

Variables	Girls	Boys	*p*-Value	Effect Size
HRQoL	47.62 (7.84)	48.88 (7.66)	0.190 ^b^	0.100
Satisfaction with life	18.73 (4.33)	19.95 (3.42)	0.026 ^b^	0.288
Physical activity level	2.75 (0.62)	2.85 (0.62)	0.185 ^a^	0.155

Note: *p*-value obtained from *t*-test for independent sample; *p*-value obtained from Mann-Whitney U. Path a = association between satisfaction with life and physical activity level; Path b = association be-tween physical activity level and HRQoL.

## Data Availability

Data will be available upon rationale request to the corresponding author.
